# A Simple and Rapid Low-Cost Procedure for Detection of Vancomycin-Resistance Genes in Enterococci Reveals an Outbreak of Vancomycin-Variable *Enterococcus faecium*

**DOI:** 10.3390/diagnostics12092120

**Published:** 2022-08-31

**Authors:** Hozan Muhammed Abdullah, Lis Høy Marbjerg, Lise Andersen, Silje Vermedal Hoegh, Michael Kemp

**Affiliations:** 1 Clinical Department, University of Southern Denmark, 5000 Odense, Denmark; 2 Department of Clinical Microbiology, Odense University Hospital, 5000 Odense, Denmark; 3 Regional Department of Clinical Microbiology, Zealand University Hospital, 4600 Koege, Denmark; 4 Department of Regional Health Research, University of Southern Denmark, 5000 Odense, Denmark

**Keywords:** *Enterococcus faecium*, real-time PCR, *vanA*/*vanB* genes, diagnostic samples, urine specimens

## Abstract

The detection of resistance to vancomycin in enterococci cultured from patients is important for the treatment of individual patients and for the prevention of hospital transmission. Phenotypic antimicrobial resistance tests may fail to detect potential vancomycin-resistant enterococci. We have developed and tested a PCR based procedure for routine screening for vancomycin-resistance genes in clinical samples with enterococci. Primary cultures from diagnostic samples reported with growth of *Enterococcus faecium* or *E. facalis* were tested for *vanA* and *vanB* genes by real-time PCR without the isolation of specific bacteria. Up to ten samples were pooled and tested in each real-time PCR reaction, with subsequent individual testing of cultures from positive pools. In a one-month test period in 2017 *vanA* gene was detected in one out of 340 urine samples with vancomycin-susceptible enterococci reported from diagnostic culture. A second test period in 2018 included 357 urine samples, and *vanA* gene was detected in samples from eight patients. Subsequently, all urine samples reported with growth of *E. faecium* during a period of one year were tested. Fifty-eight individuals were identified with enterococci, carrying the *vanA* gene not previously detected. Routine molecular testing of primary culture material from patient samples may improve the detection of hospitalized patients carrying *E. faecium* with resistance genes to vancomycin.

## 1. Introduction

*Enterococcus faecium* is a commensal bacterium in the gut of humans and animals. However, *E. faecium* may cause infections, especially in individuals with immunocompromising conditions. The bacterium can survive for prolonged periods in the environment and has an intrinsic resistance to several widely used antibiotics favors its dissemination in hospitals. Vancomycin is often used as the first choice for the treatment of infections caused by *E. faecium* due to widespread resistance to beta-lactam antibiotics. However, since the first reports of vancomycin-resistant *E. faecium* (VREfm) in the UK and France in 1986, VREfm has spread worldwide [1,2,3]. VREfm is a high priority pathogen on The World Health Organization list of organisms for which new antibiotics are most urgently needed [4].

Resistance to vancomycin in *E. faecium* is most often mediated by the *vanA* gene carried by plasmid or by the *vanB* gene incorporated into the chromosome [2]. Phenotypic tests are traditionally used for detection of resistance to vancomycin in *E. faecium* [5]. Enterococci carrying vancomycin-resistance genes may escape detection by phenotypic methods if they are outnumbered by susceptible enterococci in mixed populations of susceptible and resistant bacteria, or if resistance is not expressed phenotypically in short term cultures. *E. faecium* carrying *vanA* gene without showing in vitro resistance to vancomycin have been reported from various parts of the world [6,7,8,9,10,11] and have caused hospital outbreaks [12,13,14]. Resistance to vancomycin may be induced by exposure to vancomycin in some of these strains and they are commonly referred to as vancomycin variable enterococci (VVE) [15,16,17,18,19]. Due to the potential induction of resistance to vancomycin, VVE may be treated as VREfm with regards to hospital infection control precautions. A clone of VVE belonging to Multi Locus Sequence Type (MLST) sequence type (ST)1421, core genome MLST (cgMLST) complex type (CT)1134, have become widespread in Denmark [12]. This strain is characterized by a deletion in the *vanX* gene [7].

Introducing molecular assays detecting resistance genes as an alternative to phenotypic susceptibility testing implies some advantages. Selection, isolation, and species identification of single colonies is not needed prior to testing for resistance genes. Thus, vancomycin resistance genes may be detected in mixed bacterial cultures such as primary cultures from patient samples. Molecular methods including real-time PCR are faster than phenotypic susceptibility testing, providing same-day results that allow for early infection control interventions. Furthermore, the high sensitivity of real-time PCR allows the detection of the target in pools of samples. In populations with a low occurrence of the target, a high number of samples can be considered negative, when pools of material from the individual samples are tested negative. This may significantly reduce costs and time for large scale testing.

The purpose of this study was to provide and test a simple and inexpensive laboratory procedure for screening for vancomycin resistance genes in samples with enterococci. The procedure was tested for two one-month periods. Coincidentally, the last of the two periods occurred at the beginning of a nationwide outbreak of VVE, and it was introduced with minor modifications as a standard diagnostic procedure.

## 2. Materials and Methods

### 2.1. Standard Routine Antibiotic Resistance Testing

Routine testing of *E. faecium* isolates for resistance to vancomycin was carried out and interpreted using disk diffusion according to The European Committee on Antimicrobial Susceptibility testing [5].

### 2.2. Sample Preparation for New Procedure

Culture plates (CHROMID^®^ CPS^®^ Elite/Columbia CNA +5% sheep blood, Biomerieux, Ballerup, Denmark) from routine diagnostic culture of urine samples from patients served as sources of bacteria. All culture plates with growth of *E. faecium* with or without other bacterial species were selected for further analyses regardless of the result of phenotypic susceptibility testing. Bacteria were transferred to PCR tubes with 250 µL of sterile water by sweeping a 10 µL inoculum loop through the colonies on the culture plate. Bacteria from up to ten culture plates were added to each PCR tube. The colony material obtained was heated (99 °C for 10 min.) and tested by real-time PCR without further DNA extraction. The samples included in the pools of bacteria that were positive for *vanA* or *vanB* gene were subsequently individually tested by real-time PCR.

### 2.3. Real-Time PCR

The oligonucleotides used in real-time PCR assays are listed in Table 1. The *vanA* gene assay has previously been described by Fang et al. [20] and the enterococcus-specific 23S rRNA gene by Ludwig and Schleiferl. [21]. Primers and probes for *vanB* detection were designed by importing a partial *vanB* consensus sequence into the software Primer Express 3 (Applied Biosystem, Foster City, CA, USA). Primer and probe sequences were subjected to In Silico analysis using the custom-designed plugin Assay Validation to CLC Main Workbench (version 8.0) providing a detailed evaluation of primer and probe binding to all available sequences on Genbank^®^.

The *vanA* and *vanB* reactions were run as duplex real-time PCR and 23S as a separate mono-plex real-time PCR. The 25 µL total reactions contained 12.5 µL of Taqman FAST Universal PCR Master Mix (Applied Biosystems, Foster City, CA, USA), 1000 nM of each primer, 200 nM of the probes, and 5 µL of template DNA. The real-time PCR reactions were carried out as single determinations using 7500 FAST real-time PCR System (Applied Biosystems). The cycling parameters were as follows: 95 °C for 20 s, and 45 cycles of 95 °C for 3 sec, and 60 °C for 30 s. The results were analyzed using Sequence Detection Software v.1.4 (Applied Biosystems) and samples were regarded as positive if Ct-values were less than 40 with exponential real-time curves.

### 2.4. Characterization of Assays

The detection limits of the real-time PCR assays for *vanA* and *vanB* genes were determined by testing 10-fold serial dilutions of the *vanA* control strain-ATCC 51599 *E. faecium* and *vanB* control strain, N-AST8 *E. faecalis.* From each dilution, 100 µL was placed on an agar plate incubated at 37 °C overnight and the number of colony-forming units (CFU) was counted the next day. The real-time PCR efficacy (E) was calculated as the slope of Ct-value—log dilution graph using the formula: $\;efficacy=-1+10^{\left(-\frac{1}{slope}\right)}$.

### 2.5. Resistance Genes in Clinical Samples and Isolation of VREfm in Two Test Periods

From 9 May 2017 to 9 June 2017, 340 urine culture plates with the growth of *E. faecium* or *E. faecalis* in the laboratory were tested. From 10 August 2018 to 10 September 2018, 357 routine urine cultures with growth of the same species were tested. Samples were mostly from hospitalized patients with a few samples from non-hospitalized patients consulting general practitioners were included.

### 2.6. Routine Screening of Urine Cultures with E. faecium

The screening of cultures from urine samples with growth of *E. faecium* for *vanA* and *vanB* genes was introduced as a standard diagnostic routine procedure on 26 February 2019. Apart from the PCR reactions being carried out using a Lightcycler 480 instrument (Roche Molecular Systems Inc., Pleasanton, CA, USA) and the primary testing of individual samples, rather than bacteria pooled from several cultures from the procedure, was as described. A positive PCR result was reported to the requesting physician as enterococci resistant to vancomycin. The total number of urine samples and the number of patients with growth of *E. faecium* from 1 March 2019 to 29 February 2020 was extracted from the laboratory information system. The number of patients with urine samples with *E. faecium* carrying resistance genes was obtained from recordings in the infection control unit at the hospital.

### 2.7. Isolation and Typing of VRE from Patient Samples

Isolation of vancomycin-resistant enterococci from samples positive for *vanA* or *vanB* gene was completed using selective culture plates, CHROMID^®^ VRE, Biomerieux, Ballerup Denmark), and/or CHROMID^®^ CPS^®^ Elite plates (Biomerieux), to which 5 µg vancomycin Diatabs (Rosco Diagnostica, Taastrup, Denmark) were added.

For Whole Genome Sequencing (WGS) genomic DNA was extracted using DNeasy Blood and Tissue Kit (QIAGEN, Germantown, MD, USA) and fragment libraries were constructed using the Nextera Kit (Illumina, San Diego, CA, USA). Sequencing was completed using a Mieq instrument (Illumina) and 2 × 150 bp paired-end techniques according to the manufacturer’s instructions. MLST Sequence Type (ST) was inferred from the WGS using the MLST webserver (Version 1.7, https://cge.cbs.dtu.dk/services/MLST/ (accessed 15 June 2020)). The isolates were further typed in SeqSphere + version 6 (Ridom GmbH, Münster, Germany (http://www.ridom.de/seqsphere/ (accessed 15 June 2020)) using the cgMLST scheme of Complex Type (CT) by de Been et al. [22] for *E. faecium*. The ResFinder web server (https://cge.cbs.dtu.dk/services/ResFinder/ (accessed 15 June 2020)) was used to identify *van* genes in the assembled genome data, using a threshold of 90% minimum sequence identity and 60% minimum length identity cut-off.

### 2.8. Test of Stored Blood Isolates

A total of 319 stored blood isolates of *E. faecium* and 141 isolates of *E. faecalis* from blood cultures were tested by real-time PCR for *vanA* and *vanB* genes. The *E. faecium* blood isolates were obtained from January 2016 to April 2018 and *E. faecalis* from selected periods between October 2010 to May 2018. As for the bacteria from culture plates, up to ten blood isolates were pooled for initial testing.

### 2.9. Follow-Up on Positive Test Results in a High-Risk Clinical Department

Due to previous episodes of transmission of vancomycin-resistant *E. faecium* in a department of haematology, the detection of *E. faecium* carrying the *vanA* gene in the urine of patients in this department was followed up by screening of all patients in the department using the GeneXpert system (Cepheid, CA, USA) [23].

## 3. Results

### 3.1. Characterization of Assays

The detection limits of the real-time PCR assays without the prior extraction of nucleic acids was 3750 Colony Forming Units (CFU) per reaction for the *vanA* gene assay and 3950 CFU per reaction for the *vanB* gene. The real-time PCR efficiency was 75% for the *vanA* gene and 55% for the *vanB* gene.

### 3.2. Results from the Two Test Periods

Using the culture and PCR methods described, 340 and 357 urine samples were tested in 2017 and 2018, respectively (Table 2).

In 2017, the *vanA* gene was detected in one sample with vancomycin-resistant enterococci not reported from a standard culture. In 2018 *vanA* gene was detected in sixteen samples from which vancomycin-resistant enterococci had not been reported from a standard culture. The sixteen samples were from eight individual patients. *E. faecium* carrying *vanA* gene was isolated from five of the eight patients with samples positive for *vanA* gene in 2018 (Table 3). The bacteria had not previously been detected in any of these patients. WGS and typing confirmed the presence of the *vanA* gene and revealed that four of the five isolates belonged to ST1421-CT1134 and had a deletion in the *vanX* gene.

**Figure 1:**
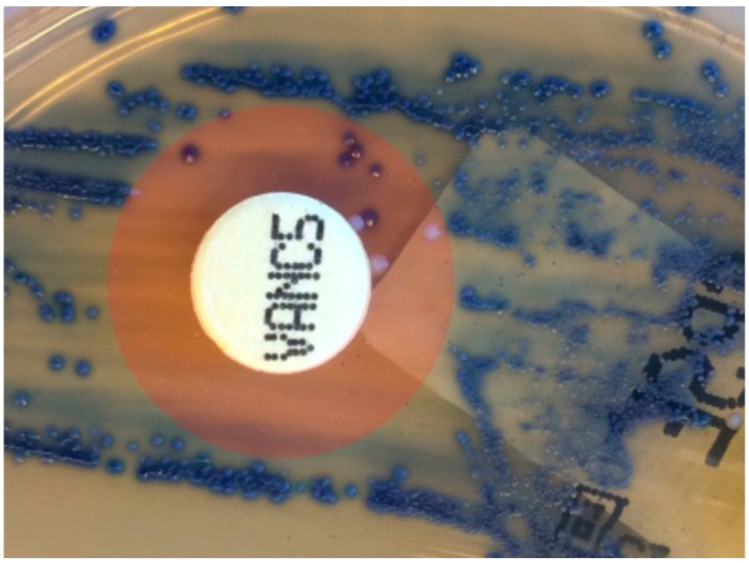
Demonstration of mixed culture of *E. faecium* susceptible and resistant to vancomycin from a urine culture. A clear inhibition zone (marked by red) for most of the bacteria is present with a few resistant colonies. *E. faecium* is blue/green on the culture medium used (CPS Elite). White colonies are contaminants.

Subculture of *E. faecium* from one urine culture revealed the presence of very few vancomycin-resistant *E. faecium* concomitant with a majority of vancomycin-resistant bacteria (Figure 1). Two colonies outside and two colonies in the inhibition zone were selected for whole genome sequencing. The colonies outside the zone were characterized as *E. faecium* ST80-CT1953 with no *van* genes detected, while the colonies from the zone were *E. faecium* ST1421-CT1134, positive for *vanA*, *vanH* and partial *vanX* genes.

### 3.3. Routine Testing of Cultures from Urine Samples

Over a one-year period *E. faecium* was cultured from 1089 urine samples from 606 patients. In 58 patients the urine samples processed as described were the first specimen from which *E. faecium* was cultured and vancomycin-resistance genes were detected. Of these samples 56 were positive for *vanA* gene, one was positive for *vanB* gene, and one sample was positive for both *vanA* and *vanB* gene. *E. faecium* carrying resistance genes to vancomycin were subsequently isolated and typed from 42 of the 58 first-time positive cultures. Thirty-nine (93%) of the isolates carrying resistance genes to vancomycin were VVE ST1421-CT1134.

### 3.4. Vancomycin-Resistance Genes in Isolates from Blood Cultures

Stored blood isolates of *E. faecium* and *E. faecalis* from the preceding years were tested for *vanA* and *vanB* genes using the same pooling protocol as described for clinical samples. No enterococci carrying unrecognized vancomycin resistance genes were detected.

### 3.5. Follow-Up on Positive Test Results in a High-Risk Clinical Department

**Figure 2:**
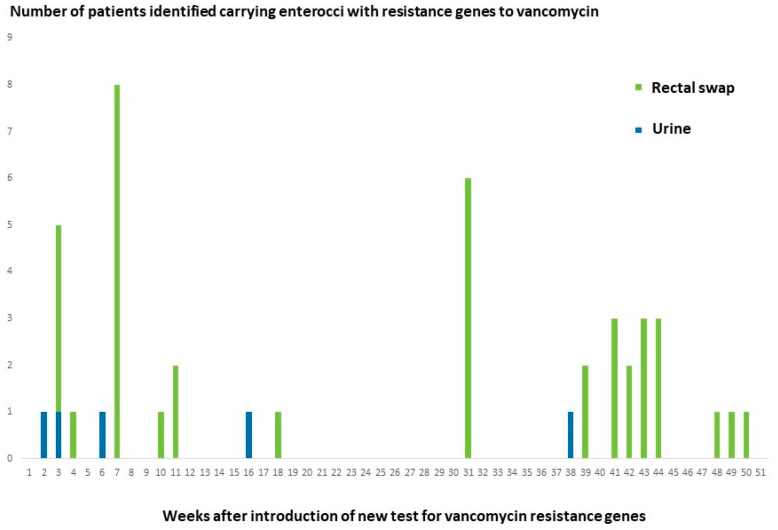
Epi-curve showing number of patients with *E. faecium* carrying resistance genes detected over a one-year period by PCR of urine cultures (Urine) and by targeted screening (Rectal swap) in a haematological department. Detection of *E. faecium* carrying *vanA* gene in the urine of a patient was followed by further identification of carriers among patients in the hospital department by screening using rectal swaps and GeneXpert. Only first time detection of *vanA* gene is included for each patient.

In a high-risk clinical department with a history of transmission of vancomycin resistant *E. faecium*, the detection of resistance genes to vancomycin in urine cultures with growth of *E. faecium* was followed up by standard rectal swap screening of other patients in the wards using the GeneXpert system. As seen in Figure 2 detection of *E. faecium* carrying *vanA* gene in diagnostic urine samples often preceded positive screening samples in other patients.

## 4. Discussion

Vancomycin-resistant *E. faecium* is a clinically important pathogen. The proportion of blood isolates of *E. faecium* with resistance to vancomycin had increased from 8.1% in 2012 to 19.0% in 2018 in Europe [24]. Studies had shown a higher mortality rate from invasive infections by vancomycin-resistant enterococci than infections by vancomycin susceptible enterococci [15]. Predisposing factors for acquiring bacteraemia by vancomycin-resistant enterococci included conditions such as diabetes mellitus and malignancies [16]. Anti-neoplastic chemotherapy and the consumption of specific groups of antibiotics, including third-generation cephalosporins, glycopeptides, and metronidazole, were also risk factors for infections by vancomycin-resistant enterococci [15]. Subclinical transmissions between hospitalized patients was well-described [17], and efficient surveillance was recommended to prevent hospital transmission of vancomycin-resistant enterococci [18].

Fearing failure of vancomycin-resistance being detected in enterococci by standard phenotypic assays, we established the procedure described here. The system is inexpensive (less than 10 EUR per PCR run including all three assays), simple, and rapid. It may be modified to fit into individual laboratory settings, for instance by using other PCR assays than the tests used in this study. In order to save time and resources, the real-time PCR assays were applied without prior DNA extraction. This affected the PCR efficacies as shown. However, lack of inhibition and a high number of target bacteria on the culture plates after incubation made this simple procedure sufficient for detection of resistance genes that were not identified by the culture-based standard procedures. In contrast to what had been reported from systems that detected resistance genes without a culture step [23,25], false positive results for *vanB* gene were not encountered in the test periods. False-positive results may have resulted from bacteria other than enterococci that carried the *vanB* gene [26]. These bacteria included anaerobes such as *Clostridium species* [27], which do not grow under the standard conditions used for routine culture of urine in this study.

In order to ensure the detection of resistance genes to vancomycin, even when these were carried by only a minority of the *E. faecium* population in the sample, the procedure applied did not include the selection of single colonies for resistance determination. As shown, we identified a case of such mixed infection during the second test period. Interestingly, the vancomycin-resistant isolate was a VVE ST1321-CT1134. This illustrated the ability of this strain to express resistance, as described previously [7].

Four of the five *E. faecium* carrying *vanA* gene during the second test period were typed as ST1421-CT1134. At that time, this type had previously only been detected in the laboratory in samples from one hospitalized patient and two patients consulting general practitioners. One of the VVE ST1421-CT1134 *E. faecium* was isolated from a patient who had been hospitalized at the same ward and at the same time as the patient previously identified with this strain. It was concluded that the transmission between patients had most likely taken place, and standard infection control interventions were initiated. Using the procedure described here, the detection of VVE ST1421-CT1134 subsequently increased rapidly [28]. Over a one year period, 93% of patients with first detection of *E. faecium* carrying resistance genes to vancomycin in urine were infected by VVE ST1421-CT1134. The VVE ST1421-CT1134 *E. faecium* detected in this period were part of a nationwide outbreak [12].

The detection of VVE ST1421-CT1134, which may not be recognized by phenotypic susceptibility testing, raised concern that this strain had been present at the hospital for a prolonged period of time without being recognized. Therefore, stored blood isolates from the preceding years were analyzed without detection of *vanA* or *vanB* genes. We believe that VVE ST1421-CT1134 has been introduced relatively recently before it was detected in four patients during the second test period. As the stored isolates had been picked from single colonies, the presence of both vancomycin-resistant and vancomycin-susceptible *E. faecium* strains in the original patient sample could not be ruled out. The importance of improved diagnostics for detecting transmission in hospitals was demonstrated by the results from follow up screening in a hospital department. As shown in Figure 2 identification of patients with urinary infection with *E. faecium* carrying *vanA* gene repeatedly resulted in the detection of other patients in the department with rectal swaps positive for *vanA* gene, demonstrating ongoing transmission.

The present study has some limitations. We did not test if the addition of a DNA extraction step would have an impact on the sensitivity and/or specificity of the analyses. As the procedure increased the detection rate considerably, we prioritized speed over possible improved sensitivity. Another limitation is the use of in-house PCR assays for detection of resistance genes in the cultures. Further studies are needed to establish the performance of other detection systems in the procedure.

## 5. Conclusions

We here present a rapid and inexpensive procedure improving detection of enterococci carrying *vanA* or *vanB* gene in clinical samples. Primary cultures were analyzed for resistance genes by specific PCR assays.

The procedure allows immediate initiation of appropriate infection control measures. Repeated routine application of the procedure for one-month periods revealed a sudden occurrence of vancomycin-variable *E. faecium* reported as susceptible to vancomycin based on phenotypic testing. Consequently, the procedure was used for more than a year, resulting in the detection of a regular outbreak of VVE. Application of the procedure at intervals or on a more permanent basis may ensure the detection of potential vancomycin-resistant enterococci escaping detection by standard methods.

## Figures and Tables

**Table 1:** Primer and probe sequences used for in-house real-time PCR assays in this study.

Gene	Oligo Sequence	Reference
vanA-F	5′-AGT CAA TAC TCT GCC CGG TTT C-3′	Fang et al. [20]
vanA-R	5′-GCA GCG GCC ATC ATA CG-3′	
vanA-P	5′-FAM-CGT CAT ACA GTC GTT ATC-MGB-3′	
vanB-F	5′-GGR AAC GAG GAT TTG ATT G-3′	This study
vanB-R	5′-CGT GGC TCA RCC GGA TT-3′	
vanB-P	5′-VIC-CGG CGA AGT GGA TC-MGB-3′	
23S-F	5′-AGA AAT TCC AAA CGA ACT TG-3′	Ludwig and Schleifer [21]
23S-R	5′-CAG TGC TCT ACC TCC ATC ATT-3′	
23S-P	5′-FAM-TGG TTC TCT CCG AAA TAG CTT TAGGGC TA-BHQ1-3′	

**Table 2:** Results from phenotypic resistance testing and subsequent identification of resistance genes and from PCR screening for resistance genes of primary cultures from urine samples with growth of enterococci in two test periods. The six vancomycin-resistant isolates in 2017 were all from cultures found positive for the same resistance genes when the primary cultures were tested by real time PCR.

Test Period	Number of Urine Samples Tested	Number of Samples Reported with Culture of Vancomycin Resistant Enterococci Based on Phenotypic Resistance Testing with Subsequent Identification of Resistance Genes by PCR	Number of Primary Cultures Positive for *vanA* or *vanB* Gene
One month from 2017	340	2 *vanA* gene, 4 *vanB* gene	3 *vanA* gene, 4 *vanB* gene
One month from 2018	357	0	16 *vanA* gene

**Table 3:** Species identification and typing results from urine samples tested positive for vancomycin resistance genes in the second (2018) test period.

Patient #	Number of Urine Samples Positive for *vanA* or *vanB* Gene	Species	MLST Sequence Type (ST) and Core Genome MLST Cluster Type (CT)
1	1 sample positive for *vanA* gene	*E. faecium*	ST1421-CT1134
2	1 sample positive for *vanA* gene	*E. faecium*	ST1421-CT1134
3	7 samples positive for *vanA* gene	*E. faecium*	ST1421-CT1134
4	2 samples positive for *vanA* gene	*E. faecium*	ST1421-CT1134
5	1 sample positive for *vanA* gene	*-*	No isolate obtained
6	1 sample positive for *vanA* gene	*-*	No isolate obtained
7	1 sample positive for *vanA* gene	*E. faecium*	ST80-CT993
8	1 sample positive for *vanA* gene	*-*	No isolate obtained

## Data Availability

The data presented in this study are available on request from the corresponding author.
